# The Use of Diethoxydimethylsilane as the Basis of a Hybrid Organosilicon Material for the Production of Biosensitive Membranes for Sensory Devices

**DOI:** 10.3390/membranes12100983

**Published:** 2022-10-10

**Authors:** Olga A. Kamanina, Elizaveta A. Lantsova, Pavel V. Rybochkin, Vyacheslav A. Arlyapov, Yulia V. Plekhanova, Anatoly N. Reshetilov

**Affiliations:** 1 Laboratory of Biologically Active Compounds and Biocomposites, Tula State University, Lenin pr. 92, 300012 Tula, Russia; 2 G.K. Skryabin Institute of Biochemistry and Physiology of Microorganisms, Pushchino Center for Biological Research of the Russian Academy of Sciences, 5 Prosp. Nauki, Pushchino, 142290 Moscow, Russia

**Keywords:** sol–gel membrane, organosilicon membrane, *Paraccocus yeei*, biocomposites, biomembrane, diethoxydimethylsilane, tetraethoxysilane

## Abstract

Biomembranes based on an organosilica sol–gel matrix were used to immobilize bacteria *Paracoccus yeei* VKM B-3302 as part of a biochemical oxygen demand (BOD) biosensor. Diethoxydimethylsilane (DEDMS) and tetraethoxysilane (TEOS) were used as precursors to create the matrix in a 1:1 volume ratio. The use of scanning electron microscopy (SEM) and the low-temperature nitrogen adsorption method (BET) showed that the sol–gel matrix forms a capsule around microorganisms that does not prevent the exchange of substrates and waste products of bacteria to the cells. The use of DEDMS as part of the matrix made it possible to increase the sensitivity coefficient of the biosensor for determining BOD by two orders of magnitude compared to a biosensor based on methyltriethoxysilane (MTES). Additionally, the long-term stability of the bioreceptor increased to 68 days. The use of such a matrix neutralized the effect of heavy metal ions on the microorganisms’ catalytic activity in the biosensor. The developed biosensor was used to analyze water samples from water sources in the Tula region (Russia).

## 1. Introduction

The creation of new materials using nanotechnologies and the improvement of their physicochemical properties are of great scientific and practical importance [1,2]. The sol–gel process is of particular interest for the production of nanostructured materials [3,4]. This approach opens up new prospects for obtaining matrices with high purity and uniformity, makes it possible to carry out various chemical processes at low temperatures and simultaneously introduce biological material into the matrix structure, which expands the scope of such hybrid biocomposites. The production of porous materials with embedded microorganism cells allows creating new types of composites with improved properties to solve environmental problems.

Immobilized cells are able to maintain their viability for a long time, which provides additional advantages when they are used in industrial and environmental biotechnology [5]. One of the modern approaches for the immobilization of microorganisms is their encapsulation. Artificial shells protect cells from physical and chemical stress factors; therefore, microorganisms in an artificial capsule are sometimes called “artificial spores”, emphasizing their similarity in this respect with natural spores of microorganisms [6]. The sol–gel method can be used to obtain such materials, since the method does not require energy-intensive, expensive equipment, it is economical and environmentally friendly, and most importantly, the sol–gel synthesis reactions proceed under mild conditions, which is important for the immobilization of the living cells [5]. The initial compounds for synthesis can be organosilica compounds—precursors (for example, alkoxysilanes and alkylalkoxysilanes) that are hydrolyzed with subsequent condensation, forming sol particles, and then a gel.

Over the past ten years, our research team has been conducting research on the production of the porous hybrid biocatalysts using sol–gel technology. The simple and mild conditions for sol–gel synthesis were proposed, under which the spontaneous formation of an organosilicate capsule around methylotrophic yeast cells [7] occurs when tetraethoxysilane and methyltriethoxysilane are used as silane precursors [8]. The processes of the utilization of wastewater containing methanol with oxygen saturation [9] and without oxygen saturation [10] have been studied. The possibility of the efficient disposal of contaminants with the developed biofilter was shown [10]. A unique approach to the encapsulation of the yeast cells was proposed [11]. Previously, we used the following substances as a structure-controlling agent: polyethylene glycol (PEG) or polyvinyl alcohol (PVA). We have shown that when using PVA, the cells retained their activity longer, and the metrological characteristics were better [9]. Therefore, PVA was used for this work.

An important component in the sol–gel synthesis of biohybrids is alkylalkoxysilanes (a hydrophobic additive, which contains non-hydrolysable bonds). Sol–gel materials are often used to form hybrid materials containing organic functional groups that are attached to an inorganic structure [12]. For this, various precursors are used that contain not only Si-OR groups that can be effectively hydrolyzed, but also Si-C bonds that are resistant to the hydrolysis [13]. As a result, the final material contains organic groups that did not participate in the sol–gel reactions. The use of this synthesis scheme makes it easy to incorporate the organic functional groups into the resulting organo-inorganic lattice. These functional groups can modify the chemical reactivity and polarity of the silica lattice, as well as impart certain optical or electronic properties of the material. A branching and an increase in the chain length of the precursor substituent reduces the rate of hydrolysis [12,14]. However, it is necessary to use alkylalkoxysilanes with non-hydrolysable Si-C bonds as precursors to create more favorable conditions for the biomaterial functioning [11].

Methyltriethoxysilane (MTES) and diethoxydimethylsilane (DEDMS) are common alkyl-substituted ethoxysilane monomers, which are often used to incorporate the silicon–carbon bond into the organo-inorganic silica structure, which leads to the preparation of new materials [15,16,17]. All previous studies of our research group were performed using a compound with one non-hydrolysable bond, methyltriethoxysilane, as a hydrophobic additive to tetraethoxysilane. Diethoxydimethylsilane contains two non-hydrolysable bonds and is the starting compound in the synthesis of silicones. It can be expected that when DEDMS is used as a precursor, a less rigid but strong shell will be formed around the cells of microorganisms. It is known [12,13,14,15] that the gelation time also increases upon passing from tetraethoxysilane (TEOS) to DEDMS, which may be due to the influence of the steric effect of precursors on the gelation rate.

Organically modified silica particles are produced from two or more precursors whose reactivity is critical to product homogeneity. The addition of a functional organic silane to TEOS leads to the production of different polymers with different structures and properties [17]. It has been proven that the final properties of the polymer depend on the stages of hydrolysis and condensation of ethoxysilanes [18,19], since they determine the subunits from which further structures are made. In the presented work, for the first time, the effect of adding DEDMS on the formation of biomembranes using living cells of microorganisms was studied.

Studies were carried out with *Paracoccus yeei* bacterial cells isolated from activated sludge from purification plants. Activated sludge microorganisms have a high biotechnological potential and unique metabolic properties—in particular, a very wide range of oxidizable substrates and a high rate of their oxidation [20,21], which makes them promising biological agents for creating hybrid biocomposites. The use of activated sludge makes it possible to increase the quantity/variety of substrates oxidized by the cells. This property is especially important in the development of biosensors for determining the biochemical oxygen demand (BOD), which is an integral characteristic of water pollution [22]. However, such sensors are unstable, and since the activated sludge biocenosis can change over time, they are characterized by low reproducibility, low operational and long-term stability. Thereby, it is more efficient to use a pure culture of microorganisms isolated from activated sludge as part of BOD biosensors. 

Therefore, the purpose of this work was to create and study the properties of a biomembrane based on an organosilicon sol–gel matrix containing DEDMS. This hybrid composite was used to immobilize cells of *Paracoccus yeei* VKM B-3302 bacteria as part of a biosensor to determine the level of BOD in various water sources.

## 2. Materials and Methods

### 2.1. Microorganism Cultivation

The *Paracoccus yeei* VKM B-3302 strain was isolated from activated sludge and characterized by the team of authors of the article together with the staff of the All-Russian Collection of Microorganisms of the Institute of Biochemistry and Physiology of Microorganisms, Pushchino [23].

### 2.2. Obtaining a Biosensor Receptor Element by Cell Encapsulation via the Sol–Gel Method

The silane precursors used were tetraethoxysilane (TEOS) and diethoxydimethylsilane (DEDMS). A mixture of silane precursors with hydrophobic additives DEDMS content 50% (vol.) with respect to the total amount of the silane precursor was used. A suspension of cells of 1.2 ± 0.1 × 10^9^ CFU/cm^3^ in phosphate buffer saline (pH 6.8) was added to 0.02 cm^3^ of a 5% solution of polyvinyl alcohol (PVA) (Ferak Berlin, Germany) in phosphate buffer saline and stirred for 5 min (Elmi CM-70M07, Riga, Latvia), then a mixture of tetraethoxysilane and diethoxydimethylsilane (SigmaAldrich, Burlington, MA, USA) of volume 0.1 cm^3^ was added and mixed again for 5 min. Then, 0.005 cm^3^ of the catalyst solution of 0.02 mol/dm^3^ NaF was added and stirred for 15 min, then placed on the surface of a Clark oxygen electrode (Econix-Expert Ltd., Moscow, Russia).

### 2.3. Measuring the Catalytic Activity of Cells in a Biosensor

The multifunctional analyzer thermooximeter–multimeter Expert-001 (Ekoniks, Moscow, Russia) in the “thermooximeter” mode was used to record and convert the signal. The signal was recorded continuously. The device was controlled using the built-in program "EXP3RT". The system was washed with sodium–potassium phosphate buffer solution (4 cm^3^, 0.02 mol/dm^3^, pH 6.8) before measurements and between measurements. When the substrate was introduced into the measuring cuvette, the oxygen consumption by the cells increased during the oxidation of the substrate, due to which the oxygen concentration in the near-electrode space decreased. Therefore, the recorded parameter was the rate of change in the output signal of the oxygen electrode, which is proportionally related to the rate of change in the concentration of dissolved oxygen in the near-electrode space. The change in oxygen consumption by cells was proportional to the concentration of the applied substrate.

### 2.4. Sample Pore Structure Study by Low-Temperature Nitrogen Adsorption Method (BET Method)

The samples were successively washed by shaking in distilled water and ethanol for 15 min at a speed of 150 rpm to bring them to a single standard. Then the samples were dried in a heat chamber for 120 min at 200 °C. Then, 0.1–0.3 g of the standardized sample was put in a weighed quartz cuvette. The cuvette was placed into a sample preparation device Beckman Coulter M SA-PREPTM (Beckman Coulter, Pasadena, CA, USA), where it was dried using ultrapure nitrogen (99.999%, GOST 9293–74) for 60 min at a temperature of up to 60 °C. The prepared sample was placed in a port of Beckman Coulter M SA 3100TM (Beckman Coulter, USA) for gas removal. All gas was removed within 60 min under a pressure of 0.001 mm Hg at 60 °C. After that, the sample in a cuvette was cooled and weighted to determine its precise weight (±0.0005 g). Then, it was put into an analysis port of an SA 3100^TM^ device to measure its surface properties (specific surface area, pore structure) based on nitrogen adsorption at a constant temperature. Pore structure (micro-, meso- or macroporous) was studied under relative pressure ranging from 0 to 0.995 mm Hg.

### 2.5. Scanning Electron Microscopy

Scanning electron microscopy (SEM) analysis was performed using a JSM-6510 LV (JEOL, Tokio, Japan) scanning electron microscope in a low vacuum (30 Pa) mode to register backscattered electrons (BSE), and in a high vacuum mode to register secondary electrons (SE).

### 2.6. Energy Dispersive X-ray Spectroscopy and Elemental Mapping

A target-oriented approach was utilized for the optimization of the analytic measurements [24]. Before measurements, the samples were mounted on a 25 mm aluminum specimen stub and fixed by graphite adhesive tape. Metal coating with a thin film (10 nm) of gold/palladium alloy (60/40) was performed using the magnetron sputtering method as described earlier [25]. The observations were carried out using a Hitachi SU8000 (Hitachi, Tokio, Japan) field-emission scanning electron microscope (FE-SEM). Images were acquired in secondary electron mode at 5 kV accelerating voltage and at a working distance of 8-10 mm. The morphology of the samples was studied, taking into account the possible influence of the metal coating on the surface [25]. EDX studies were carried out using the Oxford Instruments X-max EDX system (Oxfords instruments plc, Abingdon, UK).

### 2.7. Determination of BOD_5_ by Standard Dilution Method

The dilution method was used as a reference method for determining BOD_5_. The analysis was carried out in accordance with the procedure specified in the federal nature conservation regulations [26]. The determination of the content of dissolved oxygen in the test samples was carried out by the amperometric method in accordance with the standard procedure. 

## 3. Results

### 3.1. Determination of the Structure of Biomembranes Obtained by Sol–Gel Synthesis

Structural features of hybrid organosilicon materials can be associated with the polymerization degree of silicates (under the conditions of basic catalysis, with primary polymerization, which occurs in parallel with hydrolysis). The degree of polymerization can be estimated from the number of Si-O-Si bonds formed by IR spectroscopy.

**Figure 1:**
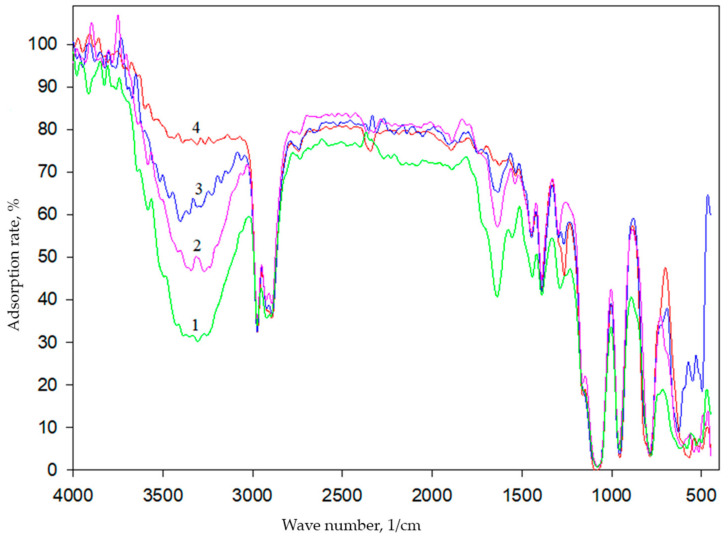
IR spectra of an organosilicon matrix obtained within an hour (1—100% TEOS, 2—85% TEOS, 3—50% TEOS, 4—15% TEOS).

The obtained spectra are similar (Figure 1) when different ratios of silane precursors are used. It is known that as the siloxane chains become longer or more branched, the absorption of Si-O-Si becomes broader and more complex, showing two or more overlapping bands [27]. As seen in Figure 1, in the IR spectra, there are characteristic absorption bands in the range of 1110–1000 cm^−1^, corresponding to vibrations of the Si-O-Si bonds. The spectra also contain an intense absorption band in the region of 1270 cm^−1^, which corresponds to the bending vibrations of Si-CH_3_ groups that are not subjected to the hydrolysis. The obtained data are in good agreement with the data described in the literature [28,29,30]. Data on the formation of sol–gel matrices using TEOS and DEDMS as silane precursors have been obtained for the first time.

**Figure 2:**
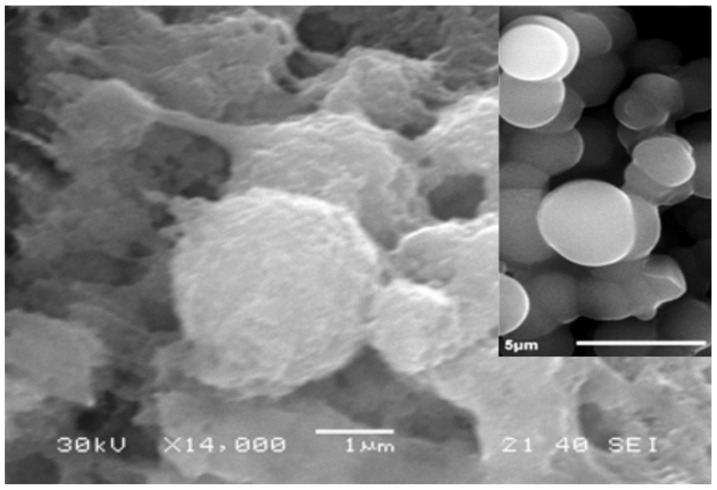
SEM results of bacteria *Paracoccus yeei* VKM B-3302 encapsulated in a sol–gel matrix (the inset shows a micrograph of free cells).

At the next stage of the work, bacterial cells were immobilized into the created matrix. *Paracoccus yeei* bacteria were isolated by members of the project team from the activated sludge of treatment facilities. Using the scanning electron microscopy, we visualized the morphology of the resulting organosilicate matrix with immobilized *Paracoccus yeei* bacteria (Figure 2).

Using scanning electron microscopy, it was shown that non-immobilized *P. yeei* bacteria are spherical cells 1–3 μm in size (inset of Figure 2). When bacteria are immobilized in a sol–gel matrix, spherical particles 4–5 μm in size are visualized, which are encapsulated cells of microorganisms. Similar structures for Gram-negative bacteria *Paracoccus yeei* were obtained for the first time.

**Figure 3:**
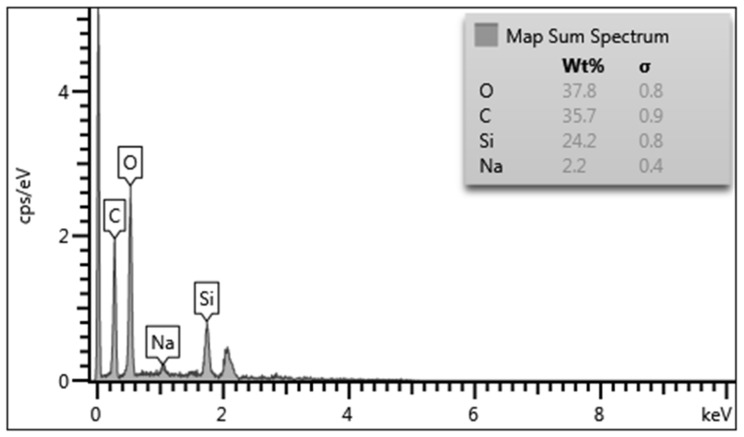
Determination of the composition of the matrix containing 50% DMDES and immobilized microorganisms.

The method of energy dispersive X-ray spectroscopy (EDS) was used to determine the elemental composition and to map the surface of the formed heterogeneous biocatalyst in the selected microdomain. According to the EDS results (Figure 3), O, C, Si and Na are the main constituent elements of the sol–gel matrix with immobilized yeast cultures.

**Figure 4:**
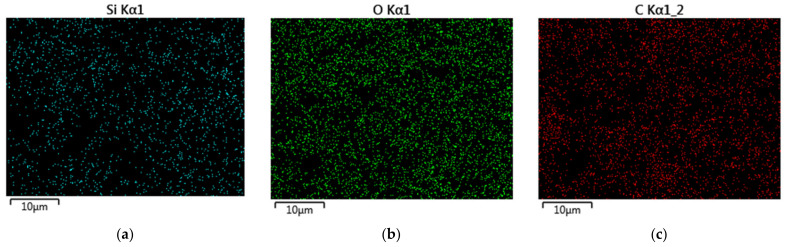
EDS elemental maps of microdomain of the sol–gel matrix containing 50% DEDMS and immobilized microorganisms: (**a**) silicon; (**b**) oxygen; (**c**) carbon.

This distribution is due to the fact that oxygen, carbon and silicon are the main elements in the composition of organosilicate shells that form around microorganisms. Additionally, oxygen is included in the composition of polyvinyl alcohol, which is a structure-controlling agent in the formation of the matrix. The high carbon content in the biohybrid material is natural due to the presence of two non-hydrolyzable alkyl radicals (CH_3_) in the 50% DEDMS matrix; moreover, PVA contains carbon. Sodium is included in the buffer (pH = 6.8), which was used to make the suspension of microorganisms. The absence of nitrogen and phosphorus on the surface of the sol–gel matrix indicates that the cells of microorganisms are reliably immobilized in the layer of the sol–gel material, which is obtained on the basis of the matrix using 50% DEDMS and 50% TEOS. The EDS maps of the sample are presented in Figure 4.

Figure 4 shows the EDS maps for the elements that are present in the developed sol–gel matrix. It can be seen from the EDS maps that the main elements, silicon and oxygen, are distributed fairly evenly over the sample surface.

**Figure 5:**
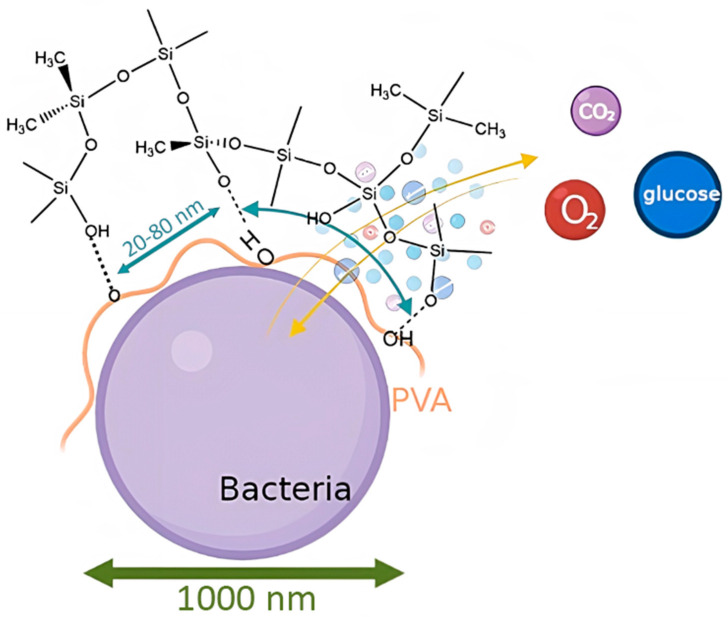
Proposed model for the formation of a sol–gel matrix around microorganism cells.

These data suggest that the organosilicon matrix forms a shell around the cells of microorganisms, which should reliably hold the cells on the electrode surface, as well as protecting the cells from division and from the harmful effects of environmental factors. The proposed matrix formation scheme is shown in Figure 5.

**Figure 6:**
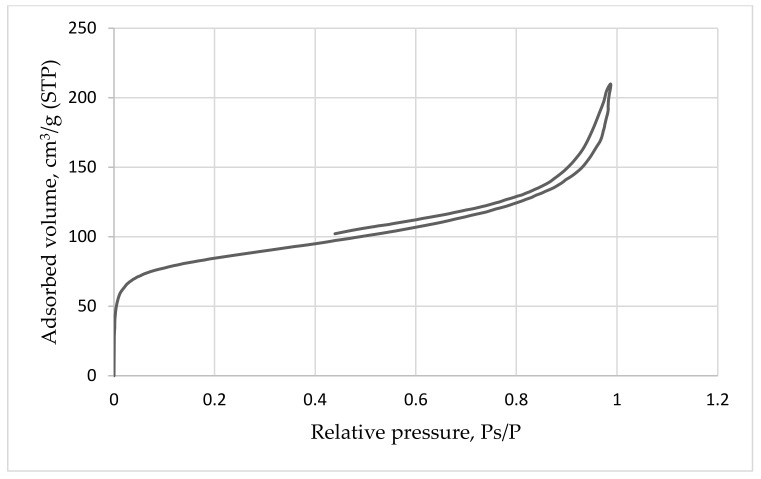
Nitrogen sorption/desorption isotherm on the sample surface.

The obtained biohybrids were analyzed by the method of low-temperature nitrogen adsorption to determine their porosity and specific surface area; the results are shown in Figure 6.

**Figure 7:**
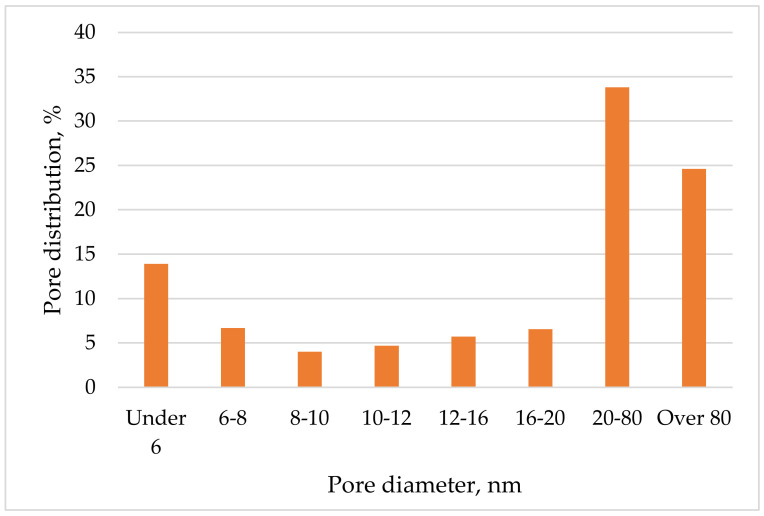
Pore distribution in the sample.

The resulting isotherm (Figure 6) belongs to type II and is typical for nonporous solids. Based on the BJH model (Barrett–Joyner–Halenda), the distribution of pores by volume was obtained for a biocatalyst based on bacteria immobilized in a sol–gel matrix with a ratio of TEOS/ DEDMS silane precursors: 50/50 (Figure 7).

As can be seen from Figure 7, the size of the largest number of pores in the sample is in the range from 20 to 80 nm, so mesopores and macropores below 100 nm in diameter predominate in the sample. Thus, bacteria (whose size is 1-2 μm) are securely fixed in the sol–gel matrix and are not able to be washed out, while their metabolic products and substrates can diffuse without restrictions through pores of such sizes.

### 3.2. Evaluation of the Catalytic Properties of the Obtained Biomembranes

Previously, we have shown that bacterial cells have the best catalytic activity when immobilized in a sol–gel matrix containing 50 vol.% hydrophobic additive (MTES) and 50 vol.% TEOS [31]. Therefore, for this work, we chose a ratio of 50 vol.% DEDMS and 50 vol.% TEOS.

**Figure 8:**
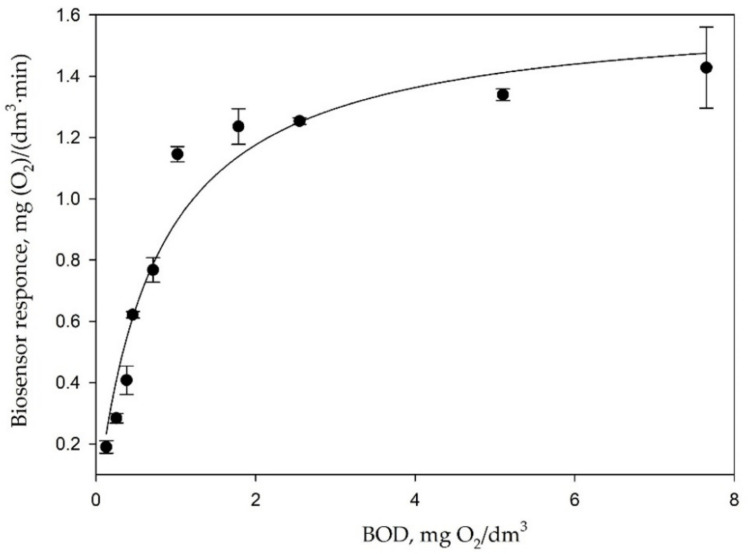
Calibration dependence of the response of a bacterial biosensor based on a sol–gel matrix to the content of biochemical oxygen demand.

The catalytic activity of encapsulated bacteria was studied, and the main analytical and metrological characteristics were determined [32], which makes it possible to judge the efficiency of the functioning of microorganisms in the sol–gel material (Table 1). The biocatalyst is based on microorganism cells, and their functioning is subject to the equation of enzymatic kinetics. The data of the calibration dependences (Figure 8) were analyzed using the Michaelis–Menten kinetics, using the equation:

(1) $$r=\frac{r_{\max\;}{\left[S\right]}_{0}}{K_{m}+{\left[S\right]}_{0}}$$

where r_max_ is the maximum rate of the enzymatic reaction; K_m_ is the Michaelis constant numerically equal to the substrate concentration at which the enzymatic reaction rate reaches half of the maximum value; S_0_ is the substrate concentration.

The constant K_m_ of this equation was taken as the upper boundary of the linear range of the calibration curve for biosensors. The sensitivity coefficient was determined as the tangent of the slope of the rectilinear section of the calibration function (Table 1). Characteristics of the biosensor obtained on the basis of *P. yeei* bacteria encapsulated in an organosilicon sol–gel matrix with a precursor ratio of 50 vol.% DEDMS and 50 vol.% TEOS were compared with the previously studied bioreceptor element based on the same bacteria, where the MTES precursor was used instead of DEDMS [7].

**Figure 9:**
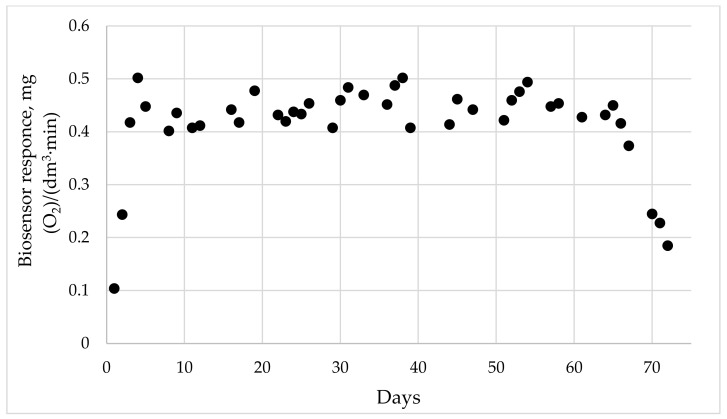
Diagram of long-term stability of biosensor.

To assess the long-term stability of cells in the biosensor, the response of the biosensor was obtained within several days. We assumed that the cells lost their activity when the biosensor response was observed to be less than 50% or more relative to the maximum response (Figure 9). In this work, we include the period of adaptation of the cells (three first days) in the long-term stability.

As can be seen from the diagram, the response of the biosensor was above 50% of the relative maximum on day 67. We concluded that the catalytic activity of the cells persisted for 67 days.

The replacement of methyltriethoxysilane [7] with diethoxydimethylsilane led to a narrower range of determined BOD contents and a lower limit of determined concentrations. Presumably, this is due to the peculiarity of the DEDMS structure, which has a smaller number of Si-O– bonds, which leads to the formation of a material with a flexible and flat structure. However, the characteristics of sensitivity, as well as long-term stability, are several times higher than the values of these characteristics for a biosensor based on MTES (Table 1). Thus, the bacteria are securely fixed in the sol–gel matrix on the electrode surface and retain their activity for 67 days.

### 3.3. Evaluation of the Protective Properties of the Obtained Biomembranes

For the practical application of the obtained biomembranes, it is necessary to study the influence of environmental factors (heavy metal ions and UV irradiation) on the stability and physiological activity of *Paracoccus yeei* bacteria immobilized in a sol–gel matrix based on TEOS and DEDMS 50/50.

The protective properties of the organosilicon sol–gel matrices with immobilized yeast cells have already been studied previously [11]. It has been shown that the material is able to protect yeast cells from exposure to a number of factors: UV radiation, the presence of heavy metal ions (exceeding the threshold limit value (TLV)), and pH changes. 

**Figure 10:**
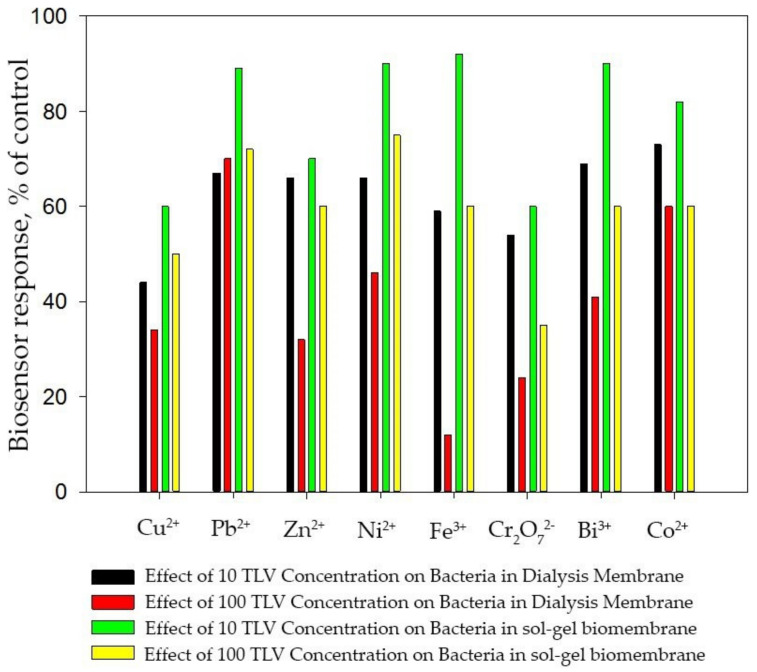
Effect of the content of heavy metal ions on the biosensor signal for the introduction of GGM using various methods of immobilization of bacteria on the electrode surface.

However, similar studies have not been carried out for cases of immobilization of bacterial cells. In order to evaluate the protective function of the matrix against metal ions, the ability of encapsulated immobilized bacteria to oxidize the glucose–glutamine mixture (GGM) in the presence of metal ions was determined (Figure 10).

Previously, the effect of metal ions on the catalytic activity of bacteria immobilized in a dialysis membrane was studied [20]. It can be seen from Figure 8 that metal ions slightly reduce the catalytic activity of microorganisms immobilized in a sol–gel matrix, even at a concentration exceeding the TLV by 100 times. Presumably, this effect is associated with the adsorption of metal ions on the surface of the sol–gel matrix.

To assess the ability of the developed matrix to protect against ultraviolet radiation, the metrological characteristics of the biosensor were compared before and after exposure to short-wave ultraviolet rays (wavelength 254 nm) for 60 min. The results are presented in Table 2.

It can be seen from the data obtained (Table 2) that the sensitivity coefficient after UV irradiation decreased by 30%, and the range of determined concentrations decreased by 41%. At the same time, after prolonged irradiation, immobilized bacteria retain a sufficiently high catalytic activity, which indicates the protective function of the organosilicon matrix. The protective properties of the matrix are related to its nature—silicate materials are impervious to short-wave (ultraviolet C) and medium-wave (ultraviolet B) UV radiation.

Thus, the developed matrix for bacterial immobilization makes it possible to protect the bioreceptor from the harmful effects of heavy metals and UV radiation, while maintaining the biosensor’s performance at a sufficiently high level for a long time.

### 3.4. Application of Obtained Biomembranes for Analysis of BOD in Real Samples

**Figure 11:**
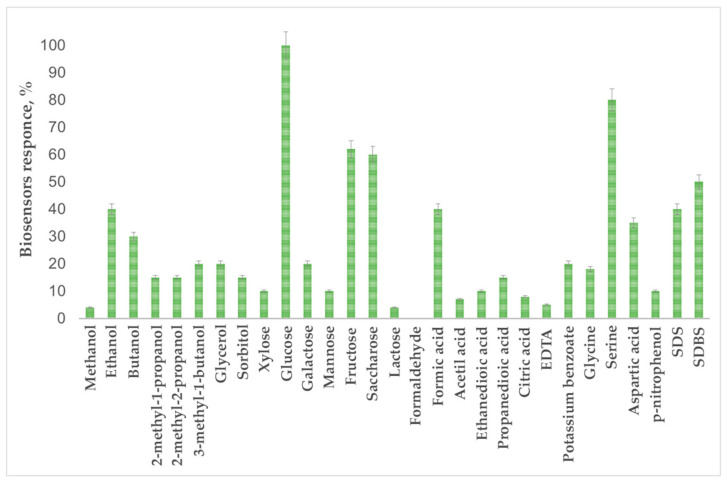
Substrate specificity of the developed biosensor.

In the case of biosensor analysis, the selectivity is determined by the substrate specificity and the method of biomaterial immobilization in the biosensor. The broad substrate specificity of the biosensor in the case of BOD determination is an advantage, as it leads to an increase in the reliability of the analysis results. In this work, we evaluated the substrate specificity of *P. yeei* bacteria immobilized in the developed organosilicon matrix for 25 substrates belonging to various classes of organic compounds. Predominantly easily oxidizable organic substances were chosen as substrates, since they are pollutants, and their entry into water bodies leads to a significant decrease in the level of dissolved oxygen and the further eutrophication of aquatic ecosystems. Bacteria oxidize substances of all presented classes of organic compounds: alcohols, carbohydrates, carboxylic acids, amino acids and nitrophenols, which can be found in waste and surface waters (Figure 11).

Previously, the substrate specificity of *P. yeei* bacteria immobilized in modified PVA was analyzed [20]. When comparing the obtained results, it can be seen that the substrate specificity profile does not fundamentally change, the sol–gel matrices are not toxic during synthesis and do not affect the enzyme systems of bacteria, and the PVA present in the hybrid material allows microorganisms to be in a comfortable environment. Therefore, the developed biomembranes can be used in the analysis of biological oxygen demand in water samples.

For the purpose of the approbation and correlative calibration of biorecognizing elements, BOD was determined in real water samples from water sources in the Tula region. The standard BOD method was used as a reference method [26]. The obtained results are presented in Table 3.

**Figure 12:**
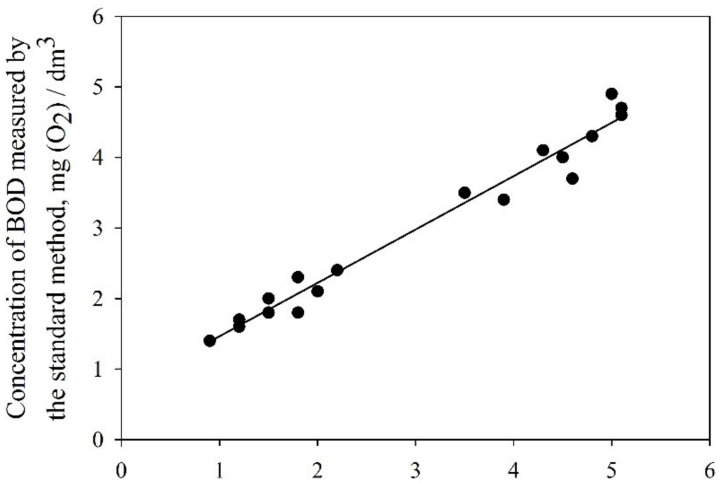
Graphical correlation of data produced by the biosensors and the standard methods.

The statistical analysis of the results of BOD determination shows that the samples obtained by two methods and for the biorecognizing element are uniform in reproducibility. The BOD values determined using the data of the BOD biosensor based on encapsulated bacteria and the standard method differ insignificantly from each other. The correlation coefficient of the data measured using the biosensor and standard methods was 0.97 (Figure 12).

Therefore, the biosensor containing the organosilicate matrix based on DEDMS and TEOS precursors (50/50 volume ratio) for immobilizing *Paracoccus yeei* bacterial cells isolated from activated sludge is a promising tool for monitoring wastewater pollution.

## 4. Conclusions

In this work, *Paracoccus yeei* VKM B-3302 bacteria were immobilized in an organosilica sol–gel matrix using DEDMS and TEOS precursors in a 1:1 volume ratio to create a biosensor bioreceptor. The SEM method showed that a capsule is formed around the microorganisms, while the BET method showed that the bacteria are securely fixed in the sol–gel matrix and are not able to be washed out, while their metabolic products and substrates easily diffuse through the pores in the obtained biomembranes.

It is shown that the replacement of MTES by DEDMS in the obtained sol–gel matrix leads to better values of such characteristics as sensitivity and long-term stability. However, a narrower range of determined concentrations characterizes the studied bioreceptor, which is associated with the specific structure of DEDMS. This precursor has fewer Si-O– bonds, which leads to the formation of a material with a flexible and flat structure. However, the sensitivity, as well as long-term stability, is several times higher than the values of these indicators for a biosensor based on the MTES precursor. In addition, the biomembrane formed as a result of the sol–gel process makes it possible to maintain a high catalytic activity of bacterial cells in the presence of heavy metal ions and UV radiation.

The developed BOD biosensor was used to analyze the degree of contamination of water samples with organic pollutants. The correlation of BOD values determined using the developed biosensors based on encapsulated bacteria and the standard method was 0.97. Therefore, DEDMS is a promising precursor for bacterial immobilization. The proposed approach to the development of a BOD biosensor based on an organosilicon sol–gel matrix with a ratio of 50 vol.% DEDMS and 50 vol.% TEOS with bacteria *Paracoccus yeei* is effective for the rapid analysis of the degree of water pollution.

## Figures and Tables

**Table 1:** Comparison of the main analytical parameters of bacterial biosensors based on *Paracoccus yeei* with various types of precursors in an immobilization matrix.

Reference		This Study	[7]
	Precursors	50 vol.% DEDMS: 50 vol.% TEOS	50 vol.% MTES: 50 vol.% TEOS
Biosensor Characteristics			
Linear BOD range, mg/dm^3^		0.076–0.800	0.1–20.0
Sensitivity coefficient × 10^−3^, min^−1^		1050 ± 90	30 ± 2
Long-term stability, days		68	31
Relative standard deviation, % (n = 10, P = 0.95)		6	3

**Table 2:** Characteristics of a biosensor based on *P. yeei* immobilized in a sol–gel matrix before and after UV irradiation.

Biosensor Characteristics	Before UV Irradiation	After UV Irradiation
Sensitivity coefficient × 10^−3^, min^−1^	1050 ± 90	750 ± 50
Minimum reporting level of determined BOD, mg/dm^3^	0.076	0.13
Upper reporting level of determined BOD, mg/dm^3^	0.80 ± 0.04	0.43 ± 0.03

**Table 3:** Determination of BOD in real samples.

№ of Water Sample	Determination of BOD, mg (O_2_)/dm^3^	
	By Biosensor Method	By the Standard Dilution Method
1	1.2 ± 0.3	1.6 ± 0.2
2	1.5 ± 0.3	2.0 ± 0.3
3	2.0 ± 0.2	2.1 ± 0.3
4	4.5 ± 0.6	4.0 ± 0.6
5	4.3 ± 0.8	4.1 ± 0.6
6	4.8 ± 0.2	4.3 ± 0.6

## Data Availability

Not applicable.

## References

[1] Liu K., Guo J., Li Y., Chen J., Li P. (2022). High-flux ultrafiltration membranes combining artificial water channels and covalent organic frameworks. Membranes.

[2] De Guzman M.R., Ang M.B.M.Y., Hsu K.-T., Chu M.-Y., Millare J.C., Huang S.-H., Tsai H.-A., Lee K.-R. (2022). Enhancing performance of thin-film nanocomposite membranes by embedding in situ silica nanoparticles. Membranes.

[3] Yandong H., Mingyong H., Wensheng Y. (2021). Sol-gel construction of mesoporous silica nanomicrostructures. Chem. J. Chin. Univ..

[4] Kamanina O.A., Saverina E.A., Rybochkin P.V., Arlyapov V.A., Vereshchagin A.N., Ananikov V.P. (2022). Preparation of hybrid sol-gel materials based on living cells of microorganisms and their application in nanotechnology. Nanomaterials.

[5] Chen S., Wang T., Yao Y., Wei A. (2018). Facile synthesis of novel fibrous silica@apatite@Au composites with superior photo-catalytic activity. Mater. Des..

[6] Hong D., Park M., Yang S.H., Lee J., Kim Y.-G., Choi I.S. (2013). Artificial spores: Cytoprotective nanoencapsulation of living cells. Trends Biotechnol..

[7] Lavrova D.G., Kamanina O.A., Machulin A.V., Suzina N.E., Alferov V.A., Ponamoreva O.N. (2017). Effect of polyethylene glycol additives on structure, stability, and biocatalytic activity of ormosil sol–gel encapsulated yeast cells. J. Sol-Gel Sci. Technol..

[8] Ponamoreva O.N., Kamanina O.A., Alferov V.A., Machulin A.V., Rogova T.V., Arlyapov V.A., Alferov S.V., Suzina N.E., Ivanova E.P. (2015). Yeast-based self-organized hybrid bio-silica sol–gels for the design of biosensors. Biosens. Bioelectron..

[9] Lavrova D.G., Kamanina O.A., Alferov V.A., Rybochkin P.V., Machulin A.V., Sidorov A.I., Ponamoreva O.N. (2021). Impact of hydrophilic polymers in organosilica matrices on structure, stability, and biocatalytic activity of immobilized methylotrophic yeast used as biofilter bed. Enzym. Microb. Technol..

[10] Kamanina O.A., Lavrova D.G., Arlyapov V.A., Alferov V.A., Ponamoreva O.N. (2016). Silica sol-gel encapsulated methylotrophic yeast as filling of biofilters for the removal of methanol from industrial wastewater. Enzym. Microb. Technol..

[11] Sakkos J.K., Mutlu B.R., Wackett L.P., Aksan A. (2017). Adsorption and Biodegradation of Aromatic Chemicals by Bacteria Encapsulated in a Hydrophobic Silica Gel. ACS Appl. Mater. Interfaces.

[12] Wang H., Wang Z., Liu G., Cheng X., Chi Z., Madzak C., Liu C., Chi Z. (2020). Genetical surface display of silicatein on *Yarrowia lipolytica* confers living and renewable biosilica–yeast hybrid materials. ACS Omega.

[13] Ismail W.N.W. (2016). Sol–gel technology for innovative fabric finishing—A Review. J. Sol-Gel Sci. Technol..

[14] Wang F., Liu J., Luo Z., Zhang Q., Wang P., Liang X., Li C., Chen J. (2007). Effects of diethoxydimethylsilane addition on the sol–gel process of tetraethylorthosilicate. J. Non. Cryst. Solids.

[15] Yildirim N., Odaci D., Ozturk G., Alp S., Ergun Y., Dornbusch K., Feller K.-H., Timur S. (2011). Sol–gel encapsulated glucose oxidase arrays based on a pH sensitive fluorescent dye. Dye. Pigm..

[16] Habib O., Demirkol D.O., Timur S. (2012). Sol–gel/chitosan/gold nanoparticle-modified electrode in mediated bacterial biosensor. Food Anal. Methods.

[17] Irani M., Keshtkar A.R., Moosavian M.A. (2012). Removal of cadmium from aqueous solution using mesoporous PVA/TEOS/APTES composite nanofiber prepared by sol–gel/electrospinning. Chem. Eng. J..

[18] Liu R., Xu Y., Wu D., Sun Y., Gao H., Yuan H., Deng F. (2004). Comparative study on the hydrolysis kinetics of substituted ethoxysilanes by liquid-state ^29^Si NMR. J. Non. Cryst. Solids.

[19] Schmidt H., Scholze H., Kaiser A. (1984). Principles of hydrolysis and condensation reaction of alkoxysilanes. J. Non. Cryst. Solids.

[20] Arlyapov V.A., Yudina N.Y., Asulyan L.D., Kamanina O.A., Alferov S.V., Shumsky A.N., Machulin A.V., Alferov V.A., Reshetilov A.N. (2020). Registration of BOD using *Paracoccus yeei* bacteria isolated from activated sludge. 3 Biotech.

[21] Arlyapov V.A., Kharkova A.S., Kurbanaliyeva S.K., Kuznetsova L.S., Machulin A.V., Tarasov S.E., Melnikov P.V., Ponamoreva O.N., Alferov V.A., Reshetilov A.N. (2021). Use of biocompatible redox-active polymers based on carbon nanotubes and modified organic matrices for development of a highly sensitive BOD biosensor. Enzym. Microb. Technol..

[22] Febriasari A., Huriya, Ananto A.H., Suhartini M., Kartohardjono S. (2021). Polysulfone–polyvinyl pyrrolidone blend polymer composite membranes for batik industrial wastewater treatment. Membranes.

[23] Kharkova A.S., Arlyapov V.A., Turovskaya A.D., Avtukh A.N., Starodumova I.P., Reshetilov A.N. (2019). Mediator BOD biosensor based on cells of microorganisms isolated from activated sludge. Appl. Biochem. Microbiol..

[24] Kachala V.V., Khemchyan L.L., Kashin A.S., Orlov N.V., Grachev A.A., Zalesskiy S.S., Ananikov V.P. (2013). Target-oriented analysis of gaseous, liquid and solid chemical systems by mass spectrometry, nuclear magnetic resonance spectroscopy and electron microscopy. Russ. Chem. Rev..

[25] Kashin A.S., Ananikov V.P. (2011). A SEM study of nanosized metal films and metal nanoparticles obtained by magnetron sputtering. Russ. Chem. Bull..

[26] (2003). Water Quality—Determination of Biochemical Oxygen Demand after N Days (BODn), Part 1: Dilution and Seeding Method with Allylthiourea Addition.

[27] Arkles B., Arkles B., Larson G.L. (2013). Infrared analysis of organosilicon compounds. Silicon Compounds: Silanes & Silicones.

[28] Pereira A.P.V., Vasconcelos W.L., Oréfice R.L. (2000). Novel multicomponent silicate–poly(vinyl alcohol) hybrids with controlled reactivity. J. Non. Cryst. Solids.

[29] Shokri B., Abbasi-Firouzjah M., Hosseini S.I. FTIR analysis of silicon dioxide thin film deposited by Metal organic-based PECVD. Proceedings of the 19th International Symposium on Plasma Chemistry Society.

[30] Kohler T., Hejtmann G., Henneck S., Schubert M., Guyenot M. (2022). Sol–gel encapsulation for power electronics utilizing 3-Glycidyloxypropyltriethoxysilane and 3-Mercaptopropyltrimethoxysilane. J. Sol-Gel Sci. Technol..

[31] Kamanina O.A., Arlyapov V.A., Rybochkin P.V., Lavrova D.G., Podsevalova E.A., Ponamoreva O.N. (2021). Application of organosilicate matrix based on methyltriethoxysilane, PVA and bacteria *Paracoccus yeei* to create a highly sensitive BOD. 3 Biotech.

[32] Thévenot D.R., Toth K., Durst R.A., Wilson G.S. (2001). Electrochemical biosensors: Recommended definitions and classification. Biosens. Bioelectron..

